# Secretome of pleural effusions associated with non-small cell lung cancer (NSCLC) and malignant mesothelioma: therapeutic implications

**DOI:** 10.18632/oncotarget.27290

**Published:** 2019-11-05

**Authors:** Albert D. Donnenberg, James D. Luketich, Vera S. Donnenberg

**Affiliations:** ^1^University of Pittsburgh School of Medicine, Department of Medicine, Pittsburgh, PA, USA; ^2^UPMC Hillman Cancer Centers, Pittsburgh, PA, USA; ^3^McGowan Institute for Regenerative Medicine, Pittsburgh, PA, USA; ^4^University of Pittsburgh School of Medicine, Department of Cardiothoracic Surgery, Pittsburgh, PA, USA

**Keywords:** pleural effusion, non-small cell lung cancer, malignant mesothelioma, cytokines, IL-6

## Abstract

Introduction: We compared the secretome of metastatic (non-small cell lung cancer (NSCLC)) and primary (mesothelioma) malignant pleural effusions, benign effusions and the published plasma profile of patients receiving chimeric antigen receptor T cells (CAR-T), to determine factors unique to neoplasia in pleural effusion (PE) and those accompanying an efficacious peripheral anti-tumor immune response.

Materials and Methods: Cryopreserved cell-free PE fluid from 101 NSCLC patients, 8 mesothelioma and 13 with benign PE was assayed for a panel of 40 cytokines/chemokines using the Luminex system.

Results: Profiles of benign and malignant PE were dominated by high concentrations of sIL-6Rα, CCL2/MCP1, CXCL10/IP10, IL-6, TGFβ1, CCL22/MDC, CXCL8/IL-8 and IL-10. Malignant PE contained significantly higher (*p* < 0.01, Bonferroni-corrected) concentrations of MIP1β, CCL22/MDC, CX3CL1/fractalkine, IFNα2, IFNγ, VEGF, IL-1α and FGF2. When grouped by function, mesothelioma PE had lower effector cytokines than NSCLC PE. Comparing NSCLC PE and published plasma levels of CAR-T recipients, both were dominated by sIL-6Rα and IL-6 but NSCLC PE had more VEGF, FGF2 and TNFα, and less IL-2, IL-4, IL-13, IL-15, MIP1α and IFNγ.

Conclusions: An immunosuppressive, wound-healing environment characterizes both benign and malignant PE. A dampened effector response (IFNα2, IFNγ, MIP1α, TNFα and TNFβ) was detected in NSCLC PE, but not mesothelioma or benign PE. The data indicate that immune effectors are present in NSCLC PE and suggest that the IL-6/sIL-6Rα axis is a central driver of the immunosuppressive, tumor-supportive pleural environment. A combination localized antibody-based immunotherapy with or without cellular therapy may be justified in this uniformly fatal condition.

## INTRODUCTION

The pleura represent a common site of metastasis for non-small cell lung cancer (NSCLC) and are the primary site of tumorigenesis in malignant mesothelioma. These conditions are accompanied by the development of pleural effusions, accumulations of serous fluid rich in tumor cells, mesothelial cells, immune cells, and the cytokines and chemokines which they secrete. The pleural space is a sequestered local environment formed by mesothelial cells joined by tight junctions [1]. The levels of endogenously produced cytokines and chemokines such as IL-6 and soluble tumor necrosis factor receptor can be orders of magnitude greater in the pleura than in the plasma [2, 3]. The movement of proteins from the plasma to the pleural space is impeded to a lesser extent and is inversely related to their molecular weight [4]. Pleural effusions are not limited to malignancies and can occur when the regulation of pleural fluid volume is disrupted by pathologic changes affecting the mechanical movement of the thoracic cage, the pulmonary or systemic circulations or lymphatic drainage [5]. Benign effusions, such as those accompanying congestive heart failure or asbestosis, provide an informative contrast to malignant effusions and may reveal both the factors conditioning a site for metastasis or tumorigenesis, and differences in the pleural secretome specific to malignancy or secreted by tumor cells themselves. In this report we compare levels of 40 cytokines and chemokines (hereafter referred to as cytokines) in non-small cell lung cancer (NSCLC), mesothelioma and benign pleural effusions, with the goal of identifying druggable targets and interventions that can change the pleural environment from one that suppresses immunity and promotes tumor invasiveness, to one conducive to anti-tumor effector responses [6].

## RESULTS

**Figure 1:**
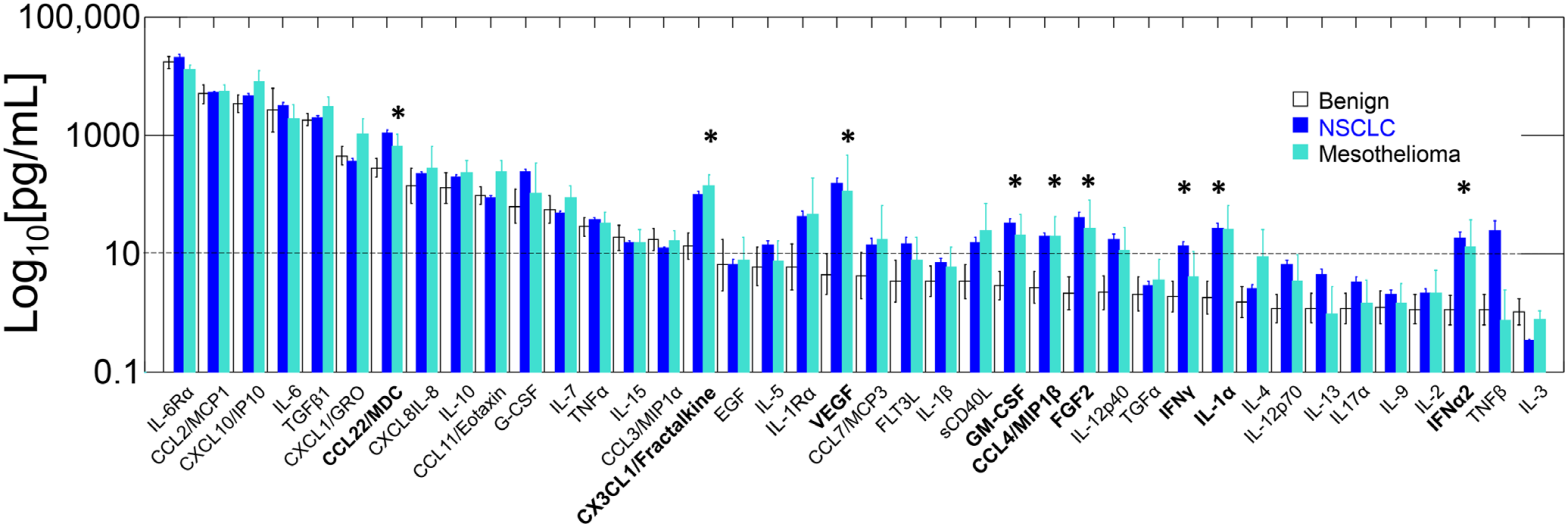
Geometric mean cytokine levels in benign, NSCLC and mesothelioma PE. Cytokines are ordered left to right from the highest to the lowest concentration in benign PE. Error bars indicate standard errors of the geometric means. Significant differences (*p* < 0.01, Bonferroni-corrected 2-tailed t-test, benign *vs* neoplastic) are shown with asterisks and bold-text. A dashed line is provided at 10 pg/mL for reference.

Figure 1 and Supplementary Table 1 show the cytokine profiles of pleural effusions from patients with malignant effusions (non-small cell lung cancer and mesothelioma) and benign effusions (heart failure, asbestos exposure without malignancy). There were no significant differences in single cytokine levels between NSCLC and mesothelioma samples (Figure 1, Student’s t-test, Bonferroni corrected for multiple comparisons), or between heart failure and asbestosis samples (not shown). The concentrations of 9 cytokines were significantly higher in malignant PE compared to benign (Supplementary Table 1). Three of the malignant PE-associated cytokines were chemoattractive (CCL4/MIP1β, CCL22/MDC, CX3CL1/ fractalkine), two were effector (IFNα2, IFNγ), one endothelial (VEGF), one inflammatory (IL-1α) and one mesenchymal (FGF2) (Table 1). Of the 6 cytokines present in concentrations greater than 1 ng/mL, only one (CCL22/MDC) was present at significantly higher concentration in malignant PE. The other cytokines highly concentrated in both malignant and benign PE were inflammatory (IL-6, IL-6Rα, CCL2/MCP1), regulatory (TGFβ1) and chemoattractive (CXCL10/IP10).

**Table 1:** Classification of measured cytokines by function

Chemoattractive	Recruit immune cells to tumor site	CXCL10/IP10, **CCL4/MIP1β, CCL22/MDC**, CXCL1/GRO, CCL11/Eotaxin, **CX3CL1/Fractalkine**, CCL7/MCP3
Effector	Anti-tumor immunity or cytotoxic functions	**IFNα2, IFNγ**, CCL3/MIP1α, TNFα, TNFβ
Endothelial	Promote angiogenesis	**VEGF**
Epithelial	Favors epithelial tumor phenotype	EGF
CCL3/Inflammatory	Elicit systematic inflammation and autoimmunity	**IL-1α**, IL-1β, IL-6, IL-6Rα, IL-17α, CCL2/MCP1
Mesenchymal	Favors mesenchymal tumor phenotype	**FGF2**
Regulatory	Dampen anti-tumor immune response	IL-4, IL-10, IL-13, TGFβ1, sCD40L, IL-1Rα
Stimulatory	Stimulation/proliferation of immune cells	**GM-CSF**, TGFα, G-CSF, Flt3L, IL-2, IL-5, IL-7, CXCL8/IL-8, IL-9, IL-12p40, IL-12p70, IL-15, IL-3

Cytokines significantly different between malignant and benign PE (Supplementary Table 1) are shown in bold. Cytokine classifications are modified from Rossi *et al*. [7].

**Figure 2:**
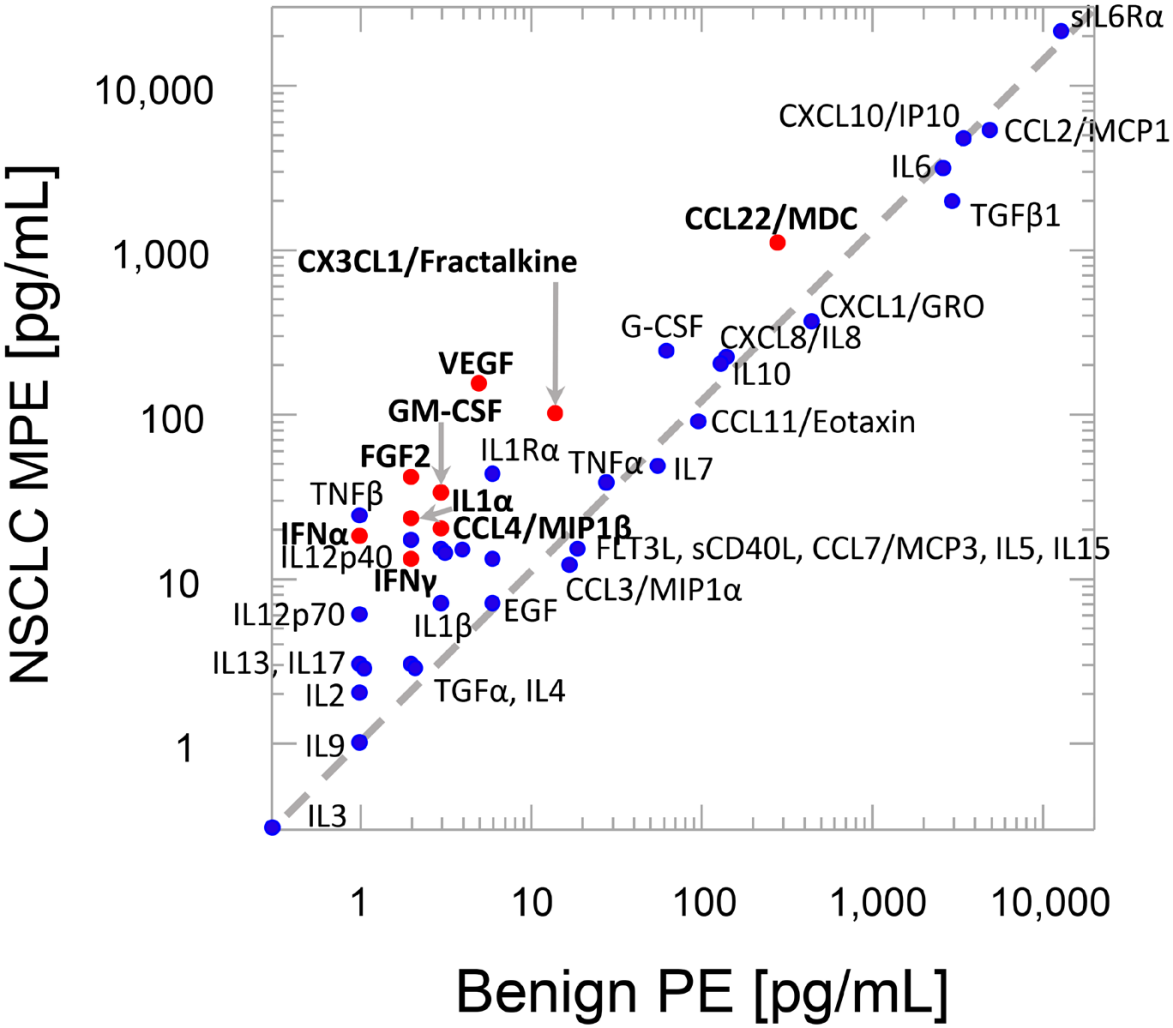
Geometric mean cytokine levels. Red symbols, bold text indicates *P* < 0.01 Bonferroni corrected 2-tailed t-test of log analyte concentrations (see Supplementary Table 1). Diagonal line indicates equal analyte concentration in benign and malignant effusions.

Figure 2 plots the geometric mean cytokine content of NSCLC PE versus the cytokine content of benign PE. Cytokines above the dashed line are more concentrated in NSCLC PE. Those in which this difference attained statistical significance are shown as red symbols with bold text.

**Figure 3:**
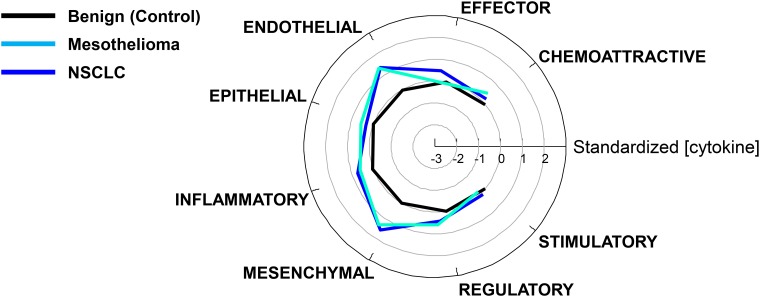
Comparison of cytokine levels grouped by function. Cytokine concentrations were standardized such that the geometric mean control value in the benign PE group was 0 with a standard deviation of 1 and grouped by function (Table 1).

Table 1 classifies the cytokine analytes into 8 functional categories as modified from a scheme proposed by Rossi et al [7]. It should be noted that some cytokines are pleiotropic and therefore could have been placed into more than one category. For example, IL-6 [8, 9], CXCL8/IL-8 [10] and TGFβ1 [11] all promote the epithelial to mesenchymal transition (EMT). In Figure 3, log cytokine values were standardized to put them on a common scale relative to control (benign) values, where 0 is the mean value of benign pleural effusions (control), and each unit represents one standard deviation from the control mean. In most functional cytokine groups, NSCLC and mesothelial PE were indistinguishable from each other and highly significantly different from benign PE (*p* < 0.002, all comparisons, Bonferroni corrected). The exceptions were the epithelial cytokine group (*F*-test not significant) and the effector cytokine group, where NSCLC was higher than benign (*p* = 0.00001, by ANOVA Tukey’s honest significant difference test), and NSCLC was higher than mesothelioma (*p* = 0.013), but mesothelioma was indistinguishable from benign (*p* = 0.7).

## DISCUSSION

### IL-6 as a key driver of the pleural immune environment

The secretome of benign pleural effusions has important implications for the biology of NSCLC cancer metastatic to the pleura and malignant mesothelioma originating in the pleural space. Little is known concerning the constitutive cytokine content of normal pleural fluid, the volume of which is small (8.4 ± 4.3 mL per side) and tightly regulated [12]. The present study demonstrates that when the homeostatic balance between fluid filtration and removal is mechanically perturbed by cardiac insufficiency or by inflammation associated with asbestosis, the resulting effusions contain a rich mixture of highly concentrated cytokines (Figures 1 and 2, Supplementary Table 1). We identified sixteen cytokines present in benign effusions at concentrations > 10 pg/mL and five exceeding 1 ng/mL. These highly concentrated cytokines are dominated by inflammatory factors (IL-6, soluble IL-6Rα/CD126, CCL2/MCP1) but also include the chemotactic factor CXCL10/IP10 and the regulatory cytokine TGFβ1. Of these, the most striking are IL-6 (mean = 2.6 ng/mL, Supplementary Table 1) and sIL-6Rα (13.0 ng/mL). IL-6 has been termed a pleiotropic cytokine because it can mediate a wide variety of pro- and anti-inflammatory effects, including stimulation or inhibition of cell growth and differentiation depending on the target cell and the environment [13, 14]. Our results agree with those of Dore et al., who reported high levels of IL-6 and sIL-6Rα in patients with malignant, benign, and infectious PE [3]. Interestingly, IL-6 and sIL-6Rα levels were higher in effusions than in plasma [3]. This argues for local production of IL-6 by mesothelial cells [15], mesenchymal cells [16], and infiltrating lymphocytes, neutrophils and macrophages. The large difference between intrapleural and systemic cytokine levels [3] reinforces the notion that the pleural space forms a distinct immune environment in which locally secreted large molecules are concentrated in isolation from the peripheral circulation.

Although the signal transducing portion of the IL-6 receptor (IL-6Rβ/CD130) is widely expressed, only leukocytes, skeletal muscle and hepatocytes normally express the complete receptor (the ligand binding protein IL-6Rα plus the signal transducing protein IL-6Rβ) and are therefore constitutively responsive to IL-6 [17, 18]. T cell receptor-activated CD4+ T cells [19], neutrophils and macrophages [20] are capable of shedding soluble IL-6Rα and are its likely source in benign PE. In the presence of sIL-6Rα, IL-6 is transformed from a highly restricted to a highly promiscuous cytokine, capable of interacting with all cell-types that express IL-6Rβ—this process has been termed trans-signaling [21]. In acute responses IL-6 participates in the resolution of neutrophil infiltration and initiation of immune effector responses, but IL-6 also increases the mononuclear cell infiltrate and participates in the pathogenesis of chronic inflammation [17].

### IL-6 in malignant PE

The importance of the IL-6/IL-6Rα axis in the pathogenesis of MPE comes from its key role as an upstream cytokine in a wound healing cascade that amplifies macrophage recruitment and promotes angiogenesis and macrophage polarization. IL-6 downregulates macrophage secretion of IL-1β, TNFα, and IL-12. It also amplifies recruitment of macrophages, T cells and dendritic cells (DC) by promoting secretion of the chemoattractant CCL2/MCP-1 by macrophages [17]. IL-6 also facilitates trans-signaling to a wide range of target cells by inducing sIL-1Rα secretion by neutrophils and macrophages. For example, endothelial and mesenchymal cells, secrete CCL2/MCP-1 in the presence of IL-6 and sIL-1Rα [17]. IL-6 may also trans-signal to epithelial cells, as it does in the thymus, upregulating CXCL1/GRO, CCL2/MCP-1, and CXCL8/IL-8 [22], three abundant cytokines in both malignant and benign PE (Figure 1). CXCL1/GRO upregulation is of particular importance in malignant PE because it is implicated in angiogenesis and tumorigenesis [23]. The central role of IL-6 trans-signaling in pathologic processes mediated by multi-cytokine cascades is further supported by the efficacy of the anti-IL-6Rα antibody tocilizumab in the treatment of rheumatoid arthritis [24] and cytokine release syndrome (CRS) induced by administration of therapeutic chimeric antigen receptor T cells (CAR-T) [25].

The present study confirms earlier reports of high IL-6 in mesothelioma PE [26] and of IL-6 plus IL-6Rα in NSCLC [27, 28] PE. The combination of IL-6 and IL-6Rα has been shown to drive VEGF production in mesothelioma cells [29]; in the present study VEGF was significantly elevated in malignant PE as compared to benign PE (Supplementary Table 1, Figure 2), suggesting a contribution by tumor cells. In NSCLC, elevated EGFR activity is associated with induction of IL-6 transcription [30], which in turn drives autocrine IL-6 signaling and tumor proliferation [31]. Interestingly, despite its tissue restriction in normal tissue, IL-6Rα has been detected in lung cancer cells and is associated with the EMT/cancer stem cell phenotype [32]. In primary adenocarcinoma of the colon, IL-6R expression has been positively correlated with EMT-associated mesenchymal markers SNAIL, SLUG, VIM, ZEB1, and ZEB2 [33]. Similarly CXCL8/IL-8, which is expressed in benign and malignant PE is associated with the mesenchymal phenotype in lung cancer [34]. We and other have shown that IL-6 and CXCL8/IL-8 play a critical role in the epithelial to mesenchymal transition of human carcinoma cells [9, 10, 35]. Taken together, the secretomes of both benign and malignant effusions are comprised of a complex mixture of cytokines dominated by IL-6 and sIL-6R. Such an environment would predictably present a protected and fertile setting for the growth and EMT of neoplastic cells.

### Other cytokines contributing to a wound-healing, immunosuppressive environment in malignant PE

We identified high levels of the regulatory cytokine TGFβ1 in both malignant and benign PE. TGFβ1 is secreted by activated platelets and is an early player in the wound healing response, directly promoting angiogenesis [36] and EMT [37]. The ability of TGFβ to amplify its own production by inducing expression in its target cells may explain why this wound healing cytokine is deleterious in chronic injury settings like cancer [38]. Indeed, high plasma levels of TGFβ1 is predictive of poor prognosis in NSCLC [39]. Further, as an immunoregulatory cytokine, TGFβ has been shown to drive naïve CD4+ T cells to differentiate into immunosuppressive regulatory T cells (T-regs) [40], an effect that may synergize with CCL4/MIP1β, which has been shown to recruit T-regs [41]. CCL22/MDC (significantly higher in MPE than benign PE, Supplementary Table 1, Bonferroni corrected *p* = 0.0028) is secreted by macrophages and DC; also attracts T-regs [42], further contributing to the suppressive immune environment of malignant PE. Like TGFβ1, high plasma levels of FGF2 are associated with shorter survival time in NSCLC [43]. This may be due to FGF2-mediated promotion of an undifferentiated state in neoplastic cells, as it does for cultured embryonic stem cells [44]. FGF2 also promotes angiogenesis indirectly by induction of VEGF secretion [45]. In NSCLC, G-CSF has been shown to enhance myeloid-derived suppressor cell function and may contribute to tumor progression [46]. G-CSF is produced by mesothelial cells in response to EGF or TNF [47], both of which are present at low levels in PE. Similarly, high levels of GM-CSF (significantly higher in MPE than benign PE, Bonferroni corrected *p* = 0.0002) are associated with lung cancer progression and invasion [48].

### A nascent immune effector response in NSCLC PE

Despite the extreme immunosuppressive, angiogenic and tumor promoting environment of the injured pleural space, there is evidence of a nascent but thwarted effector response in NSCLC, but not in malignant mesothelioma (Figure 3). This difference may be due to the fact that mesothelioma has one of the lowest mutational burdens (~3 mutations/Mb) of any cancer [49]. CXCL10, also known as IFNγ-inducible protein 10 (IP10) plays a role in the generation of effector T cells and T cell trafficking [50] and is a potent inhibitor of angiogenesis [51]. CCL2/MCP1 promotes effector responses by recruiting monocytes, memory T cells, and DC to sites of inflammation produced by tissue injury and infection [52, 53]. Both CXCL10 and CCL2 were present at high concentration in malignant and benign effusions (Figure 1, Supplementary Table 1), supporting the recruitment of potential effector cells to the pleural space. CXC3CL1/fractalkine is produced by endothelial cells and can also promote effector responses by attracting and capturing CD4+ T cells [54]. CXC3CL1 was significantly higher in NSCLC PE compared to benign PE and has been proposed as an amplification circuit of polarized Th1 responses [55]. This effect may be dampened by sIL-6Rα [56]. Most importantly, the potent effector cytokines IFNα2, IFNγ, MIP1α, TNFα and TNFβ were all detected in NSCLC PE. IFNα2 and IFNγ levels were relatively low, but significantly higher than in benign PE (Bonferroni corrected *p* = 0.0092 and 0.027, respectively), indicating a weak, yet detectable effector response. Indeed, in patients with primary NSCLC, increased plasma levels of effector cytokines within the first 3 months of diagnosis are significantly correlated with improved response to anti-PD-1 therapy and prolonged overall survival [57].

### Comparison with the cytokine environment of patients receiving CAR-T

**Figure 4:**
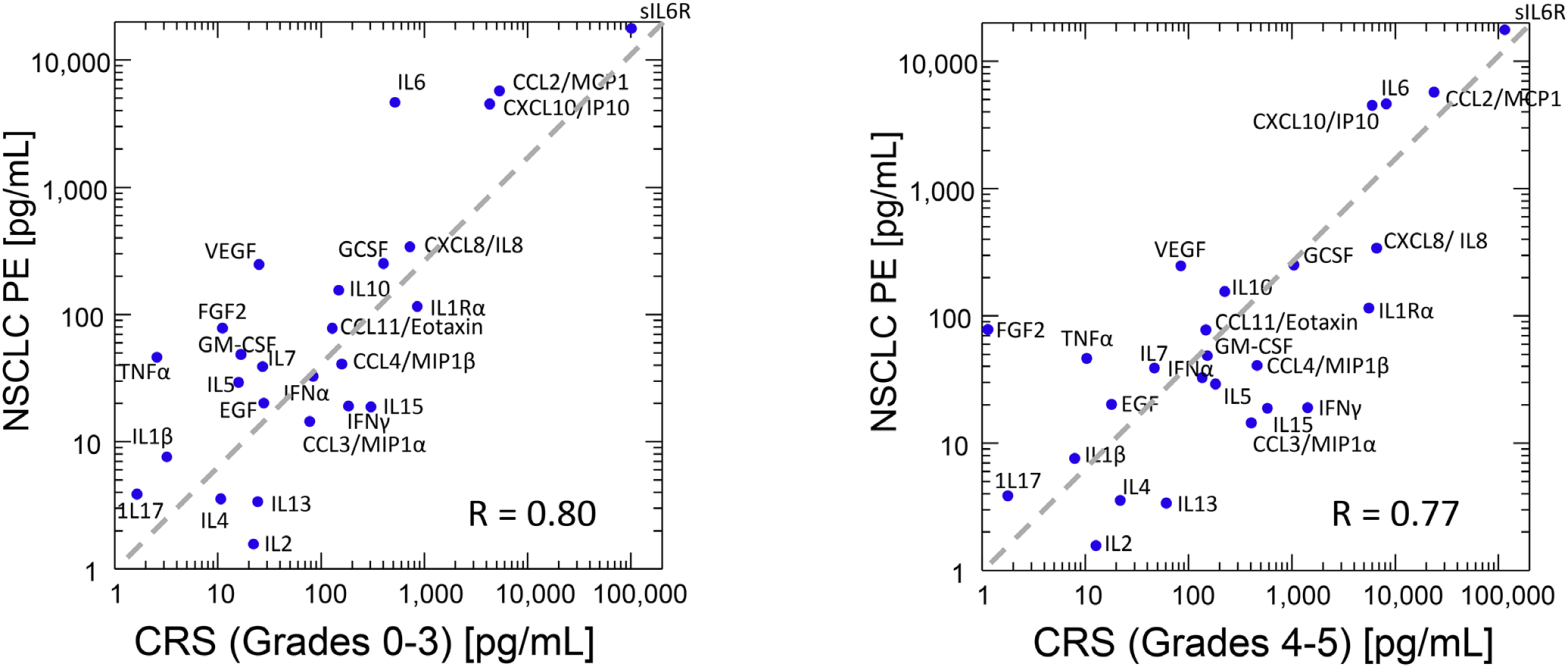
Median values of 26 cytokines common to our dataset and the median one-month-peak values published as an online supplement by Teachey *et al*. (2016). Cytokine Release Syndrome (CRS) grades 0–3, *N* = 9, CRS grades 4 and 5, *N* = 3. Pearson product-moment correlation coefficients (R), are shown. Diagonal line indicates equal analyte concentration in the plasma of CAR-T recipients and in malignant effusions.

The plasma cytokine profile elicited during successful CAR-T therapy provides a paradigm for efficacious anti-tumor effector response. Comparison of our findings with those of Teachey et al. [58], who used similar methods in patients receiving CAR-T for B-cell malignancies, is instructive. Twenty-six cytokines were shared by our respective data sets and are plotted in Figure 4. Overall, the cytokines profiles of NSCLC PE and plasma from CAR-T recipients were remarkably similar and dominated by sIL-6Rα, CCL2/MCP1, CXCL10/IP10, IL-6 and CXCL8/IL-8 (R = 0.80 and 0.77 compared to patients with CRS grades 0–3 and CRS 4–5, respectively). Compared to plasma from patients with no or minimal CRS (Figure 4, left panel), the NSCLC PE had lower concentrations of IL-2, IL-4, IL-13, IL-15, MIP1α, and IFNγ, including higher concentrations of IL-6, VEGF, FGF2, and TNFα. A similar pattern was noted when our data were compared to results from patients with clinically significant CRS (Figure 4, right panel), for which the anti-IL-6R antibody tocilizumab is an effective treatment. Importantly, despite its efficacy against CRS, tocilizumab does not appear to interfere with the expansion or efficacy of CAR-T effector cells [59]. Taken together, our findings suggest that the IL-6/sIL-6Rα axis, which is the principal driver of CRS, may also be central to the immunosuppressive, growth promoting, wound-healing milieu common to injured pleura. In malignant PE, tumor cells, whether primary or metastatic, participate in and amplify the cytokine cascade responsible for these effects.

### Toward localized combinatorial immunotherapy

Despite this hostile environment, there is evidence in NSCLC PE of a nascent immune effector response that may be harnessed therapeutically by modifying the local pleural immune environment with antibody-based therapeutics, by *ex vivo* activation and reinstillation of pleural T cells or engineered T cells [60]. We speculate that conditioning the immune environment of the pleura will greatly increase the chances of success [6]. Adusumilli *et al.* recently reported that local administration of anti-mesothelin CAR-T in combination with systemic administration of anti-PD1 agents yielded encouraging clinical results in a proportion of patients with malignant pleural disease [61]. We suggest that such results could be enhanced by conditioning the local environment using combinatorial immunotherapies in which several mechanisms of tumor-mediated immune suppression are simultaneously targeted. Our study suggests that, in addition to PD1, such combination therapies might target IL-6Rα and VEGF, for which therapeutic antibodies are available. The routine use of tunneled pleural catheters for PE drainage [62] provides the opportunity for local administration of antibody and cellular therapeutics as well as for sequential sampling and monitoring. Local delivery of combination antibody therapy to the pleural space also has the potential to decrease total delivered dose and reduce systemic toxicities.

## MATERIALS AND METHODS

### Patients and samples

Pleural effusions (PE) were collected as anonymized medical waste under an IRB exemption (No. 0503126), or IRB approved protocol No. 16110093, under which patients consented to use of the sample and access to medical records. Pleural effusions were collected from 101 NSCLC patients, 8 mesothelioma patients and 13 patients without pleural malignancy (11 with heart failure, 2 with asbestosis). Pleural fluids were picked up from the operating room or patient hospital room by laboratory staff, transported immediately to the laboratory where they were held on wet ice, and processed within 30 minutes of receipt. Cells were first removed by centrifugation (10 min at 600 x g, 4°C), and then further clarified (10 min 1880 x g, 4°C) prior to storage at –86°C. Immediately prior to analysis for cytokines and chemokines, samples were thawed and clarified by high-speed centrifugation (3 min at 16,000 x g, Beckman Microfuge E, Cat No. 348720, Beckman Coulter) in a coldroom environment (4°C).

### Quantification of cytokines

Cytokines were quantified on the Luminex platform (Hillman Cancer Center Luminex Facility, Dr. Anna Lokshin, Director), using the Curiox LT-MX plate washer, Curiox DA-96 plates, the Luminex 200 System analyzer and xPonent data acquisition and analysis software. Six-point standard curves were run for each cytokine with each of 2 sample batches. Cytokines were measured in 5 µL of neat clarified effusion using the MILLIPLEX MAP Human Cytokine/Chemokine Magnetic Bead Panel, Premixed 38 Plex (Cat. No. HCYTMAG-60K-PX38), MILLIPLEX MAP Human TGFβ (Cat. No. TGFBMAG-64K-01) and IL-6Rα from the Human Angiogenesis/Growth Factor Panel 2 (Cat. No. HANG2MAG-12K-01) kits. The first 33 samples that we assayed were run in duplicate or triplicate and had a mean difference of 5.8% (all cytokines, Supplementary Figure 1), after which samples were run without replicates. Determinations that were designated “Out of Range Below” (*i.e*., below the limit of quantification) by the analytical software were arbitrarily filled with a value 1/10 the lowest valid measurement for that cytokine. Values designated “Out of Range Above” (*i.e.*, above the limit of quantification) were assigned the value of the highest valid measurement for that cytokine.

### Statistical analysis

Descriptive statistics and statistical comparisons (Student’s t-test, 2-tailed, ANOVA) were performed on log_10_-transformed data. *P*-values were Bonferroni-corrected for multiple comparisons. For analysis of functional characteristics of cytokines unique to malignant pleural effusions, log cytokine concentrations were expressed on a common scale (standardized) relative to control values (benign effusions). This was accomplished by subtracting the mean control value from each cytokine determination and dividing by the standard deviation of the control value. SYSTAT 13 software (San Jose, CA) was used for data analysis and graphics.

## SUPPLEMENTARY MATERIALS



## Abbreviations

ANOVA: Analysis of variance
CAR-T: Chimeric antigen receptor T cell
CC, CXC, CX3C: Chemokine motif subfamilies
EGF: Epidermal growth factor
EMT: Epithelial to mesenchymal transition
FGF2: Basic fibroblast growth factor
Flt3L: Fms-related tyrosine kinase 3 ligand
G-CSF: Granulocyte colony stimulating factor
GM-CSF: Granulocyte macrophage colony stimulating factor
GRO: Growth regulated oncogene (CXCL1)
IFN: Interferon
IL: Interleukin
IP10: IFNγ-inducible protein 10 (CXCL10)
IRB: Institutional review board
L1, L2, L7, L10, L22: Chemokine ligand subfamilies
MCP1: Monocyte chemotactic protein-1 (CCL2)
MCP3: Monocyte chemotactic protein-3 (CCL7)
MDC: Macrophage-derived chemokine (CCL22)
MIP1β: Macrophage inflammatory protein-1β (CCL4)
MPE: Malignant pleural effusion
NSCLC: Non-small cell lung cancer
PE: Pleural Effusion
sCD40L: Soluble CD40 ligand
TGFα: Transforming growth factor alpha
TGFβ1: Transforming growth factor beta 1
TNFβ: Tumor necrosis factor beta
VEGF: Vascular endothelial growth factor
