# Supplementary material for: Secretome of pleural effusions associated with non-small cell lung cancer (NSCLC) and malignant mesothelioma: therapeutic implications
Source: Oncotarget. 2019 Nov 5;10(60):6456–65. doi: 10.18632/oncotarget.27290 (PMC6849644; doi:10.18632/oncotarget.27290)
Supplement: Supplementary file 1 [file oncotarget-10-6456-s001.pdf]

Secretome of pleural effusions associated with non-small cell lung cancer (NSCLC) and malignant mesothelioma: therapeutic implications

SUPPLEMENTARY MATERIALS

Supplementary Table 1: Cytokines are ordered from highest to lowest concentration in benign PE

	Benign, *N* = 13			NSCLC, *N* = 101			Mesothelioma, *N* = 8			All CA, *N* = 109			*p*-value (Benign vs. All CA)	
**Analyte**	**Geometric mean [pg/mL]**	**LCI_{95}**	**UCI_{95}**	**Geometric mean [pg/mL]**	**LCI_{95}**	**UCI_{95}**	**Geometric mean [pg/mL]**	**LCI_{95}**	**UCI_{95}**	**Geometric mean [pg/mL]**	**LCI_{95}**	**UCI_{95}**	**2-tailed Student's *t*-test**	**(Bonferroni corrected)**
**sIL-6Rα**	17,042	10,803	26,886	21,114	16,698	26,697	13,012	9,575	17,683	20,377	16,030	25,902	NS	
**CCL2/MCP1**	4,975	2,533	9,771	5,295	4,781	5,863	5,458	3,363	8,858	5,306	4,659	6,043	NS	
**CXCL10/IP10**	3,478	1,864	6,491	4,705	4,041	5,478	8,005	3,591	17,844	4,892	4,006	5,974	NS	
**IL-6**	2,638	541	12,858	3,098	2,338	4,105	1,893	692	5,177	2,988	2,138	4,176	NS	
**TGFβ1**	1,831	1,198	2,798	1,944	1,584	2,387	2,990	1,404	6,372	2,007	1,570	2,565	NS	
**GRO**	448	226	889	362	282	465	1,027	347	3,042	391	286	534	NS	
**CCL22/MDC**	283	148	542	1,092	887	1,345	661	288	1,515	1,053	817	1,356	**0.00007**	**0.0028**
**CXCL8/IL-8**	142	40	506	220	174	278	278	57	1,357	224	161	312	NS	
**IL-10**	131	43	401	201	173	233	235	101	548	203	166	248	NS	
**Eotaxin**	97	51	186	89	79	102	243	110	534	96	81	115	NS	
**G-CSF**	63	18	223	240	195	296	102	11	963	226	158	322	0.004	NS
**IL-7**	56	21	148	48	41	56	87	37	203	50	41	61	NS	
**TNFα**	28	15	55	38	34	42	33	15	73	38	32	44	NS	
**IL-15**	19	8	46	15	13	18	15	6	39	15	12	19	NS	
**MIP1α**	17	8	39	12	11	14	16	8	34	12	11	15	NS	
**CX3CL1 / Fractalkine**	14	5	34	100	77	130	139	62	310	103	76	139	**0.000005**	**0.0002**
**EGF**	6	1	42	7	4	10	8	2	40	7	4	11	NS	
**IL-5**	6	1	25	13	9	19	7	2	32	13	8	20	NS	
**IL-1Rα**	6	1	33	43	29	64	46	4	597	43	25	76	0.00424	NS
**VEGF**	5	1	20	152	100	231	112	9	1,457	149	84	264	**0.000005**	**0.0002**
**CCL7/MCP3**	4	1	23	14	8	24	18	2	198	14	7	28	NS	
**Flt3L**	3	1	16	15	9	23	8	1	40	14	8	24	0.046	NS
**IL-1β**	3	1	11	7	5	10	3	0	18	7	5	10	NS	
**sCD40L**	3	1	12	15	10	23	25	3	176	16	10	27	0.01357	NS
**GM-CSF**	3	1	8	33	25	45	20	4	93	32	22	48	**0.000005**	**0.0002**
**CCL4/MIP1β**	3	1	9	20	15	26	20	5	82	20	13	29	**0.00006**	**0.0024**
**IL-12p40**	2	1	7	17	11	27	11	2	57	17	10	28	0.0035	NS
**FGF2**	2	1	7	41	26	64	27	4	203	39	22	70	**0.00005**	**0.002**
**TGFα**	2	1	7	3	2	4	4	1	15	3	2	4	NS	
**IFNγ**	2	1	6	13	9	18	4	1	25	12	8	18	**0.00068**	**0.0272**

**IL-1α**	2	1	6	23	15	37	26	5	142	24	14	40	**0.0002**	**0.008**
**IL-4**	2	0	5	3	2	4	9	1	61	3	2	5	NS	
**IL-9**	1	0	4	1	1	2	1	0	6	1	1	2	NS	
**IL-13**	1	0	4	3	2	4	1	0	7	2	1	4	NS	
**IL-17α**	1	0	4	3	2	5	1	0	8	3	2	5	NS	
**IL-12p70**	1	0	3	6	4	9	3	1	22	6	4	10	0.00634	NS
**IL-2**	1	0	3	2	1	3	1	0	7	2	1	3	NS	
**TNFβ**	1	0	4	24	12	52	1	0	7	19	8	45	0.01208	NS
**IFNα2**	1	0	3	18	11	30	13	2	91	18	10	32	**0.00024**	**0.0096**
**IL-3**	1	0	3	0	0	0	1	0	1	0	0	0	NS	

LCI$_{95}$, UCI$_{95}$ = lower and upper 95% confidence intervals about the geometric mean.

A	Delta/Average (%)
N of Cases	1,363
Arithmetic Mean	5.8%
Standard Deviation	38.9%

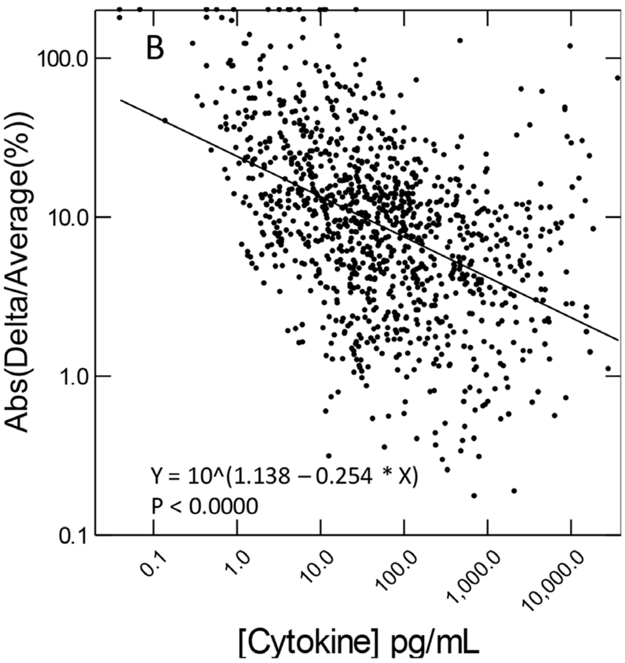

Supplementary Figure 1: Agreement between replicate cytokine determinations. A 38 cytokine/chemokine panel was run on 33 samples in duplicate or triplicate. Agreement between replicates was measured as the difference between replicates divided by their average, and expressed as a percent. (**A**) The mean difference between 1363 pairs of replicate determinations was 5.8%; (**B**) Replicate agreement was better at higher cytokine concentrations. Regression of the log absolute value of (delta/average) versus the log cytokine concentration had a highly significant negative slope ($p < 0.0000$). The least squares line of best fit is shown.
